# Explainable machine learning for predicting venous thromboembolism in septic shock patients

**DOI:** 10.3389/fimmu.2026.1860149

**Published:** 2026-07-20

**Authors:** Yuanyuan Li, Qi Xin, Yizhao Lu, Xiaoyuan Yu, Chunyu Gu

**Affiliations:** 1 Department of Emergency Surgery, Shaanxi Provincial People’s Hospital, Xi’an, China; 2 Department of Hematology, The Affiliated Hospital of Northwest University, Xi’an No. 3 Hospital, Xi’an, China; 3 Department of Emergency Medicine, Shaanxi Provincial People’s Hospital, Xi’an, China

**Keywords:** machine learning, random forest, septic shock, SHAP, venous thromboembolism

## Abstract

**Background:**

Venous thromboembolism (VTE) frequently complicates septic shock, yet precise, individualized risk stratification tools remain scarce. This study aimed to develop and externally validate an explainable machine learning (ML) framework to predict VTE in this critically ill population.

**Methods:**

A retrospective cohort study was conducted including adult septic shock patients admitted between January 2020 and December 2025. The study population was partitioned into an internal development cohort (n=733) and an independent external validation cohort from a separate tertiary hospital (n=257, Xi’an No. 3 Hospital). We utilized the Boruta algorithm alongside recursive feature elimination to isolate optimal predictors. Six ML algorithms were trained and evaluated using metrics including the area under the receiver operating characteristic curve (AUC) and F1 score. Shapley Additive Explanations (SHAP) were integrated to establish model transparency.

**Results:**

The VTE incidence within the development cohort was 17.74% (130/733). The feature selection pipeline distilled six robust predictors: fibrin degradation products (FDP), prothrombin time (PT), white blood cells (WBC), activated partial thromboplastin time (APTT), D-dimer, and C-reactive protein (CRP). Among the evaluated models, the Random Forest (RF) algorithm exhibited superior discriminative capacity and promising performance in an independent external validation cohort, achieving an AUC of 0.9718 and an F1 score of 0.7917 in the independent external validation cohort, with a sensitivity of 0.7037. SHAP analysis revealed that heightened thrombo-inflammatory markers combined with abbreviated coagulation intervals fundamentally drove VTE risk, offering personalized predictive insights via individual force plots.

**Conclusions:**

We successfully established a highly accurate and interpretable RF-based predictive model for VTE in septic shock patients. By leveraging six routine clinical biomarkers and SHAP-derived transparency, this tool bridges complex algorithmic forecasting with clinical intuition, providing a transparent risk assessment framework that may assist in risk stratification for thromboprophylaxis after prospective validation. Future implementation studies are needed to assess its real-world clinical utility and impact on patient outcomes.

## Introduction

Venous thromboembolism (VTE), which includes deep vein thrombosis (DVT) and pulmonary embolism (PE), stands as a major contributor to avoidable in−hospital morbidity and mortality around the globe (1). In Western countries, VTE is a frequent condition, with reported annual incidence rates per 100,000 population of 150 (95% CI, 131–169) for VTE, 90 (95% CI, 72–109) for DVT, and 67 (95% CI, 54–80) for PE (2). Among critically ill individuals, those suffering from septic shock face a particularly elevated risk of VTE, driven by systemic inflammatory responses, endothelial injury, hemodynamic compromise, and prolonged immobility (3–5). According to global epidemiological data, 20% to 30% of patients with sepsis in the intensive care unit (ICU) develop deep vein thrombosis (DVT) secondary to coagulopathy, and this complication is associated with a mortality rate ranging from 25% to 40% (6). Septic shock defined as sepsis accompanied by persistent hypotension requiring vasopressor therapy to maintain mean arterial pressure ≥65 mmHg despite adequate fluid resuscitation (7). Despite routine administration of pharmacological thromboprophylaxis, VTE events continue to occur in this population, underscoring the urgent need for more precise risk assessment strategies.

Early and accurate identification of VTE risk is paramount for optimizing thromboprophylaxis strategies. Currently, clinicians primarily rely on conventional scoring systems such as the Sequential Organ Failure Assessment (SOFA) and the Caprini risk score to evaluate disease severity and thrombotic risk. However, these tools may not fully capture the intricate, non-linear relationships between diverse clinical variables—such as inflammatory markers (CRP, PCT), coagulation profiles (D-dimer, FDP), and hemodynamic parameters—that drive thrombus formation during sepsis (8–10). Moreover, while standardized diagnostic protocols for DVT and PE are established, there remains a pressing need for a proactive, individualized prediction tool capable of identifying high-risk patients at an earlier stage.

Machine learning (ML) has demonstrated remarkable potential in handling high-dimensional clinical data and uncovering complex patterns that traditional statistical models might overlook. Recent years have witnessed successful applications of various ML algorithms—including logistic regression, support vector machines (SVM), random forests, gradient boosting machines, and neural networks—for VTE prediction in heterogeneous critically ill populations (11, 12). Nevertheless, the opaque nature of many ML models has hindered their acceptance in clinical practice, as physicians are hesitant to rely on predictions that cannot be explained on a per−patient basis (13, 14). To address this limitation, Shapley Additive Explanations (SHAP) has been introduced as a unified framework that quantifies each feature’s contribution to a given prediction, thereby increasing transparency and fostering trust in ML−driven decision support (15, 16).

In this retrospective study conducted at Shaanxi Provincial People’s Hospital, we aimed to develop and externally validate an interpretable ML-based framework specifically for VTE prediction in septic shock patients. By employing a robust feature selection strategy involving the Boruta algorithm and recursive feature elimination (RFE), we sought to identify the most parsimonious yet effective set of predictors. Our goal is to provide a transparent clinical tool that bridges the gap between complex algorithmic outputs and clinical intuition, ultimately facilitating personalized medical decision-making in critical care.

## Materials and methods

### Study design and population

This retrospective study was conducted at Shaanxi Provincial People’s Hospital and approved by the institutional review board (approval number: 2025R082). Written informed consent was waived due to the retrospective nature of the analysis. To assess generalizability, an independent external validation cohort was retrospectively collected from Xi’an No. 3 Hospital, a separate tertiary care center, between January 2023 and October 2025 (n=257). The use of data from Xi’an No. 3 Hospital was approved by its institutional review board (approval number: SYLL−2026−104). We screened all adult patients (aged ≥18 years) admitted with a diagnosis of septic shock between January 2020 and December 2025. Septic shock was defined according to the Sepsis−3 criteria (7), i.e., sepsis with persisting hypotension requiring vasopressors to maintain mean arterial pressure ≥65 mmHg and having a serum lactate level >2 mmol/L despite adequate volume resuscitation. Exclusion criteria were: (1) history of venous thromboembolism (VTE) before admission; (2) receipt of therapeutic anticoagulation at baseline; (3) diagnosed inherited or acquired coagulation disorders; (4) hospital length of stay <24 hours; or (5) incomplete key laboratory or outcome data.

### Data collection and variable definitions

Demographic and clinical data were extracted from the electronic medical record system, including age, sex, vital signs (temperature, heart rate [HR], respiratory rate [RR], mean arterial pressure [MAP]), comorbidities (hypertension, diabetes, chronic obstructive pulmonary disease [COPD], chronic kidney disease [CKD], cirrhosis, acute respiratory distress syndrome [ARDS], atrial fibrillation, myocardial infarction, heart failure, respiratory failure), and in−hospital interventions (continuous renal replacement therapy [CRRT], mechanical ventilation). Laboratory parameters measured within the first 24 hours of admission included coagulation profiles (prothrombin time [PT], activated partial thromboplastin time [APTT], thrombin time [TT], international normalized ratio [INR], fibrin degradation products [FDP], D−dimer [D-D], fibrinogen [FIB], prothrombin time activity [PTA]), inflammatory markers (C−reactive protein [CRP], procalcitonin [PCT]), blood cell counts (white blood cells [WBC], neutrophils, lymphocytes, monocytes, platelets, red blood cells [RBC], hemoglobin), and biochemical indices (albumin, globulin, bilirubin, alanine aminotransferase, aspartate aminotransferase, glucose, uric acid, cystatin C, blood urea nitrogen, creatinine, total cholesterol). Disease severity was assessed using the Sequential Organ Failure Assessment (SOFA) score and the Caprini risk score for VTE.

### Outcome definition

The primary outcome was the occurrence of in−hospital VTE, defined as newly diagnosed DVT or PE. DVT was confirmed by compression ultrasonography or venography in patients presenting with lower limb swelling, pain, or tenderness (1). PE was confirmed by computed tomography pulmonary angiography (CTPA) in the presence of compatible symptoms (e.g., dyspnea, tachypnea) and elevated plasma D−D levels (17).

### Handling of missing values

Due to the limitation of the sample size in this study, the data with missing values were excluded, and thus no interpolation processing was carried out on the data.

### Predictor screening strategy

To select the most relevant predictors while keeping the model parsimonious and avoiding data leakage, we performed a two−step variable selection procedure exclusively on the training set. First, the Boruta algorithm—a random forest−based method—was applied to all candidate variables; it creates shadow copies of each variable and retains only those original features whose importance is statistically greater than that of their shadow counterparts. Second, recursive feature elimination (RFE) was used to determine the optimal subset size by iteratively removing the least important variables based on the AUC. The final predictor set was chosen as the combination that achieved the highest AUC with the fewest features. To assess the stability and transportability of the selected predictors, we compared their distributions across the training, test, and internal validation cohorts using the Kruskal−Wallis test (**Table 1**). Significant inter−cohort differences would indicate population heterogeneity and inform the need for calibration before deployment in new clinical settings.

**Table 1:** Comparison of clinical data between VTE group and non-VTE group.

Variable	Total, N = 733	Non-VTE, N = 603	VTE, N = 130	*P*-value
Age	67.0 [58.0, 76.0]	66.0 [58.0, 75.0]	69.0 [61.3, 78.8]	0.028
Gender				0.033
Female	319 (43.52%)	251 (41.63%)	68 (52.31%)	
Male	414 (56.48%)	352 (58.37%)	62 (47.69%)	
Temperature	36.50 [36.20, 37.00]	36.50 [36.20, 37.00]	36.50 [36.30, 37.08]	0.413
RR	20.00 [18.00, 23.00]	20.00 [18.50, 22.00]	20.00 [18.00, 25.00]	0.279
HR	90.00 [78.00, 106.00]	89.00 [78.00, 105.00]	97.00 [83.00, 110.00]	0.010
MAP	93.33 [82.00, 103.67]	93.33 [83.00, 104.50]	92.17 [77.17, 100.58]	0.025
Hospital LOS	13.00 [8.00, 20.00]	13.00 [8.00, 20.00]	13.00 [7.00, 20.00]	0.685
Hypertension				
Yes	733 (100.00%)	603 (100.00%)	130 (100.00%)	
Diabetes				>0.999
No	85 (11.60%)	70 (11.61%)	15 (11.54%)	
Yes	648 (88.40%)	533 (88.39%)	115 (88.46%)	
COPD				0.022
No	658 (89.77%)	549 (91.04%)	109 (83.85%)	
Yes	75 (10.23%)	54 (8.96%)	21 (16.15%)	
CKD				0.047
No	432 (58.94%)	366 (60.70%)	66 (50.77%)	
Yes	301 (41.06%)	237 (39.30%)	64 (49.23%)	
Cirrhosis				0.127
No	380 (51.84%)	321 (53.23%)	59 (45.38%)	
Yes	353 (48.16%)	282 (46.77%)	71 (54.62%)	
ARDS				0.243
No	497 (67.80%)	415 (68.82%)	82 (63.08%)	
Yes	236 (32.20%)	188 (31.18%)	48 (36.92%)	
Atrial fibrillation				0.776
No	529 (72.17%)	437 (72.47%)	92 (70.77%)	
Yes	204 (27.83%)	166 (27.53%)	38 (29.23%)	
Myocardial infarction				0.393
No	686 (93.59%)	567 (94.03%)	119 (91.54%)	
Yes	47 (6.41%)	36 (5.97%)	11 (8.46%)	
Heart failure				0.428
No	675 (92.09%)	558 (92.54%)	117 (90.00%)	
Yes	58 (7.91%)	45 (7.46%)	13 (10.00%)	
Respiratory failure				0.463
No	283 (38.61%)	237 (39.30%)	46 (35.38%)	
Yes	450 (61.39%)	366 (60.70%)	84 (64.62%)	
PTA	86.10 [77.30, 92.00]	86.60 [77.00, 92.00]	86.10 [80.05, 91.00]	0.766
TT	17.00 [16.10, 18.60]	17.00 [16.10, 18.50]	17.10 [16.00, 19.05]	0.646
INR	1.24 [1.12, 1.45]	1.23 [1.12, 1.43]	1.24 [1.04, 1.52]	0.522
FDP	5.20 [1.85, 18.77]	5.10 [1.89, 13.67]	11.29 [1.78, 38.56]	0.009
D-D	1.39 [0.50, 5.90]	1.10 [0.50, 5.00]	4.30 [0.30, 15.32]	<0.001
FIB	4.58 [3.14, 6.03]	4.58 [3.13, 6.10]	4.60 [3.17, 5.69]	0.834
AAPT	39.30 [33.70, 45.00]	40.00 [35.25, 45.70]	33.30 [21.60, 41.18]	<0.001
PT	14.90 [13.30, 16.60]	15.00 [13.70, 16.70]	13.35 [1.30, 16.00]	<0.001
ALP	92.00 [70.00, 136.00]	92.00 [70.00, 132.00]	98.00 [73.25, 161.50]	0.204
Globulin	25.10 [22.00, 29.10]	25.20 [22.20, 29.15]	24.95 [20.53, 29.00]	0.505
Albumin	29.90 [25.60, 33.70]	30.40 [26.10, 34.10]	27.60 [25.13, 31.73]	<0.001
Bilirubin	14.90 [9.40, 27.30]	15.20 [9.50, 28.24]	13.35 [9.18, 23.13]	0.127
ALT	29.00 [19.00, 61.00]	29.00 [19.95, 61.00]	29.00 [18.25, 61.75]	0.976
AST	26.00 [15.00, 49.00]	26.00 [14.00, 50.50]	26.50 [17.25, 44.75]	0.819
Glucose	7.22 [5.36, 10.69]	7.18 [5.22, 10.70]	7.75 [5.88, 10.28]	0.397
Uric acid	327.0 [225.0, 469.0]	328.0 [225.5, 469.0]	319.0 [211.3, 470.3]	0.965
Cystatin C	1.63 [1.06, 2.92]	1.57 [1.05, 2.85]	2.05 [1.33, 3.29]	0.010
BUN	10.75 [6.19, 20.38]	10.20 [5.92, 19.27]	13.79 [7.42, 23.10]	0.005
Cr	111 [62, 274]	103 [62, 259]	141 [68, 363]	0.056
TC	2.92 [2.16, 3.71]	2.93 [2.18, 3.73]	2.81 [2.10, 3.57]	0.177
PCT	3.19 [0.48, 19.36]	2.51 [0.30, 16.00]	8.00 [1.34, 45.10]	<0.001
Monocyte	0.42 [0.26, 0.67]	0.42 [0.26, 0.66]	0.45 [0.31, 0.76]	0.218
RDW SD	46.20 [42.80, 51.30]	46.10 [42.65, 50.90]	46.65 [43.63, 53.93]	0.011
RDW CV	13.90 [13.10, 15.10]	13.80 [13.10, 15.05]	14.20 [13.40, 15.88]	0.025
MPV	10.80 [9.90, 11.90]	10.90 [9.85, 11.95]	10.80 [9.90, 11.88]	0.972
CRP	54.2 [10.0, 142.0]	37.0 [10.0, 119.9]	112.6 [46.5, 251.2]	<0.001
NEUT	8.10 [4.54, 13.42]	7.92 [4.35, 13.23]	8.77 [5.41, 14.54]	0.075
Lymphocyte	0.73 [0.44, 1.13]	0.75 [0.45, 1.14]	0.67 [0.39, 1.08]	0.256
WBC	9.31 [7.80, 12.72]	8.79 [7.80, 11.87]	10.78 [7.99, 27.86]	<0.001
PLT	148.0 [93.0, 215.0]	147.0 [93.5, 212.0]	149.0 [90.3, 236.8]	0.888
Hemoglobin	107.0 [88.0, 128.0]	109.0 [91.0, 129.0]	99.0 [80.0, 117.8]	<0.001
RBC	3.59 [2.91, 4.22]	3.66 [3.02, 4.27]	3.26 [2.55, 3.82]	<0.001
CRRT				<0.001
No	598 (81.58%)	511 (84.74%)	87 (66.92%)	
Yes	135 (18.42%)	92 (15.26%)	43 (33.08%)	
Mechanical ventilation				0.006
No	581 (79.26%)	490 (81.26%)	91 (70.00%)	
Yes	152 (20.74%)	113 (18.74%)	39 (30.00%)	
SOFA	7.00 [5.00, 9.00]	7.00 [5.00, 9.00]	8.00 [6.00, 10.00]	<0.001
Caprini	7.00 [5.00, 9.00]	7.00 [5.00, 8.00]	8.00 [6.00, 9.00]	<0.001

### Machine learning model construction

Six different machine learning algorithms were built to forecast VTE risk: Logistic Regression (LR), Support Vector Machine (SVM), Multilayer Perceptron (MLP), Light Gradient Boosting Machine (LightGBM), Extreme Gradient Boosting (XGBoost), and Random Forest (RF). The internal development cohort was randomly split into a training subset (70%, n = 513) and a testing subset (30%, n = 220) using stratified random sampling to maintain the original outcome proportion. The external validation cohort (n = 344) was kept untouched until final evaluation. Hyperparameters for each algorithm were tuned via five−fold cross−validation coupled with grid search on the training set, selecting the parameters that yielded the highest cross−validated AUC. All models were implemented in Python (version 3.12.5) using the scikit−learn, XGBoost, and LightGBM libraries.

Hyperparameter tuning was performed using a five−fold cross−validation combined with grid search on the training set. The final hyperparameters for each model were selected based on the highest cross−validated AUC. All models were implemented using Python 3.12.5 with the scikit−learn, XGBoost, and LightGBM libraries. The best parameters for each model are as follows: LR (C: 10, penalty: l2, solver: saga); RF (max_depth: 10, min_samples_split: 10, n_estimators: 200); SVM (C: 1, gamma: scale, kernel: linear); XGB (learning_rate: 0.1, max_depth: 3, n_estimators: 100, subsample: 0.8); LightGBM (boosting_type: gbdt, learning_rate: 0.01, n_estimators: 200, num_leaves: 31); MLP (hidden_layer_sizes: 50, learning_rate_init: 0.001, max_iter: 200). The optimal parameter configurations obtained through Grid Search effectively mitigated the risk of overfitting and enhanced the predictive performance of each machine learning model on the dataset.

The final Random Forest model was configured with 200 trees, a maximum depth of 10, a minimum of 10 samples for node splitting, and 2 samples for leaf nodes, with bootstrap sampling and balanced class weights applied. Complete hyperparameter specifications for all models are provided in **Supplementary Table 2**.

### Handling of class imbalance and classification threshold

The incidence of VTE in the development cohort was 17.74%, representing moderate class imbalance. Rather than applying synthetic balancing techniques (e.g., SMOTE, ADASYN) or undersampling, we prioritised model robustness and external validity. The Random Forest algorithm was selected partly for its inherent resilience to class imbalance, and all models were evaluated using AUC and F1 score, which are appropriate metrics for imbalanced outcomes. The default classification threshold of 0.5 was used for all models, as threshold optimisation on the training set may not generalise to external cohorts. A complementary two−step workflow (**Supplementary Figure 1**) employing SIRS/SOFA scores was developed as a clinical safety net to capture false−negative cases without altering the model’s internal threshold.

### Performance assessment metrics

Model performance was evaluated from three angles: discriminative ability, calibration, and clinical usefulness. Discrimination was measured by the AUC, accuracy, sensitivity, specificity, precision, and F1 score. Calibration was examined using calibration plots and the Brier score (a lower value indicates better agreement between predicted probabilities and actual outcomes). Clinical utility was appraised by decision curve analysis (DCA), which estimates the net benefit across different threshold probabilities. All metrics were calculated on the training set (with cross−validation), the internal test set, and the external validation cohort to assess both reproducibility and generalizability. Confidence intervals (95%) for all performance metrics were estimated using bootstrap resampling with 1,000 iterations on the external validation cohort, using the percentile method. Detailed results including CIs for all models and metrics are provided in **Supplementary Table 3**. Calibration was assessed using calibration intercept, calibration slope, and Brier score.

### Model interpretability via SHAP

To make the final model’s predictions interpretable, we applied Shapley Additive Explanations (SHAP). SHAP values break down each prediction into contributions from individual features, allowing global interpretation (summary plots showing the direction and magnitude of each feature’s effect) as well as local interpretation (force plots for individual patients). Feature importance rankings from SHAP were compared with those from logistic regression to check for consistency. For comparison, feature importance rankings from logistic regression were derived using the absolute values of standardized coefficients.

### Statistical analysis

Continuous data are reported as median with interquartile range (IQR), and comparisons between groups were performed using the Mann−Whitney U test. Categorical variables are presented as counts and percentages, with differences assessed by the chi−square test or Fisher’s exact test as appropriate.

All tests were two−sided, and a *P*−value < 0.05 was considered statistically significant. Statistical analyses were conducted using SPSS (version 27.0, IBM Corp.) and Python 3.12.5.

## Results

### Baseline characteristics of the study population

**Figure 1:**
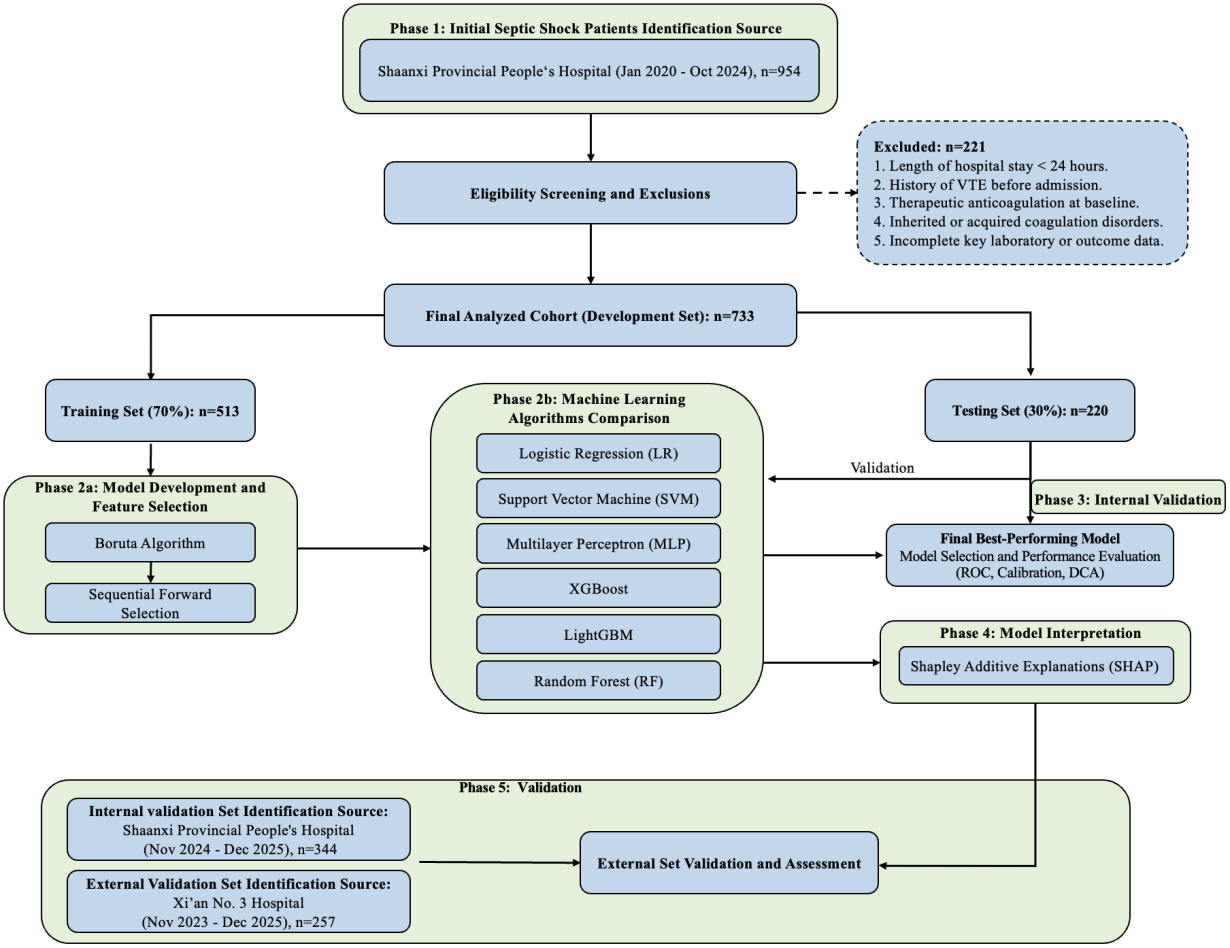
Workflow of participant enrollment, cohort stratification, and data partitioning.

A total of 733 patients with septic shock were included in this study, among whom 130 (17.74%) developed VTE. The participant flow diagram is displayed in **Figure 1**. The comparison of clinical characteristics between the VTE and non-VTE groups is summarized in **Table 1**.

Patients in the VTE group were significantly older (median 69.0 vs. 66.0 years) and had a higher proportion of female individuals (52.31% vs. 41.63%). Hemodynamic parameters such as HR and MAP also differed significantly between the two groups. In terms of comorbidities, COPD and CKD were more prevalent in the VTE group.

Notably, several laboratory markers exhibited pronounced differences. The VTE group demonstrated elevated levels of fibrin FDP, D-D, PCT, CRP, WBC, BUN, cystatin C, and RDW. In contrast, APTT, PT, albumin, hemoglobin, and RBC count were significantly lower in the VTE group. Additionally, the VTE group had higher SOFA and Caprini scores (both *P* < 0.001), and more frequently required CRRT and mechanical ventilation.

### Feature selection and variable importance

**Figure 2:**
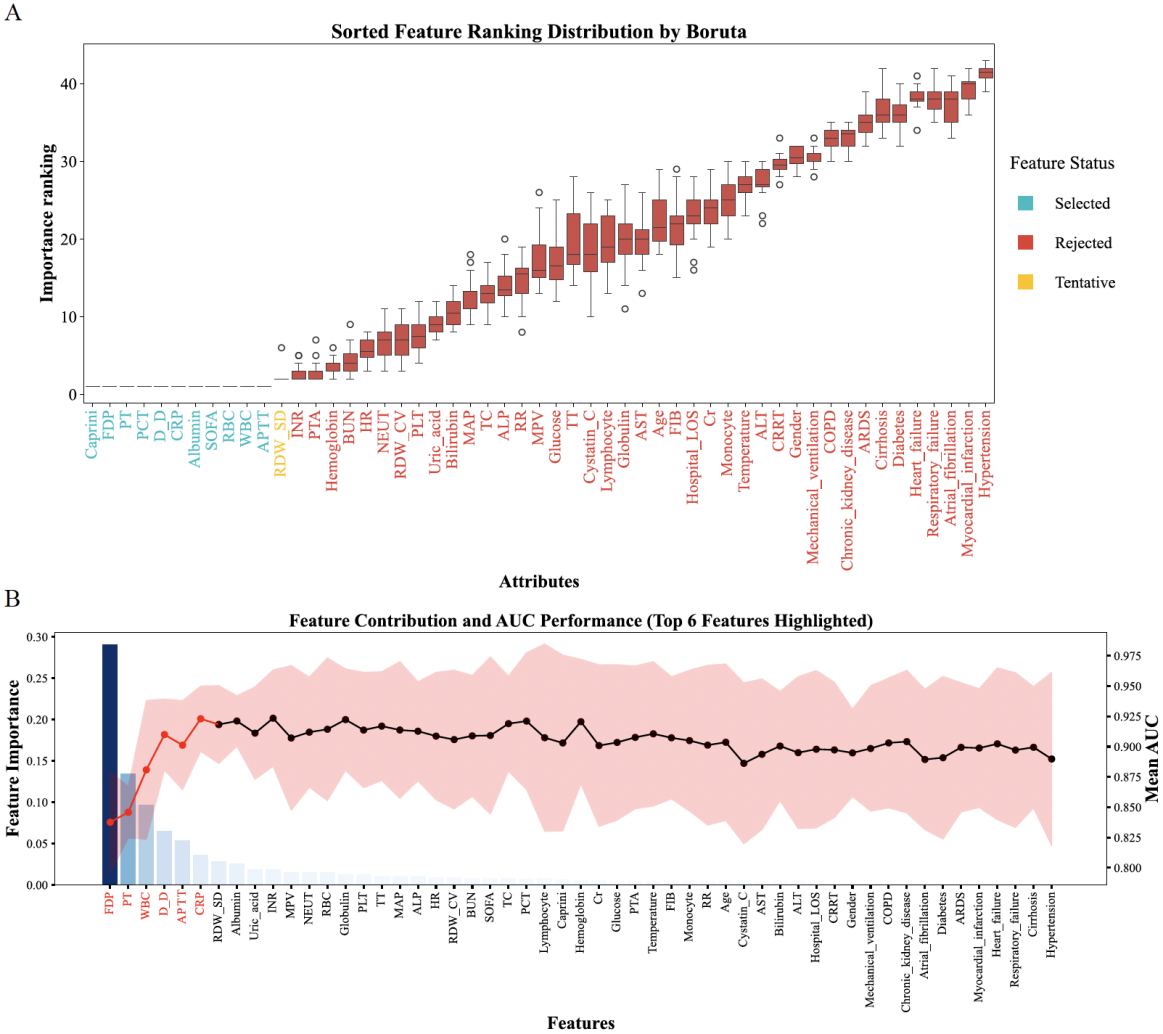
Predictor screening for VTE among septic shock patients. **(A)** Boruta importance ranking. Green boxplots represent confirmed important variables; yellow indicates uncertainty; red denotes unimportant variables. **(B)** Recursive feature elimination: bars show variable contribution; line indicates cumulative AUC; red bars denote the final six selected predictors. ALB, albumin; Hb, hemoglobin; RDW, red cell distribution width; PLT, platelets; NEUT, neutrophils; LYM, lymphocytes; MON, monocytes; GLB, globulin; ALT, alanine aminotransferase; AST, aspartate aminotransferase; ALP, alkaline phosphatase; TC, total cholesterol; UA, uric acid; BUN, blood urea nitrogen; Cr, creatinine; CysC, cystatin **(C)** Full variable names are provided in the Methods.

To identify the most relevant predictors while maintaining model parsimony, we applied the Boruta algorithm combined with recursive feature elimination. As shown in **Figure 2**, the Boruta analysis confirmed multiple important variables (green boxplots), predominantly coagulation markers (D−dimer, FDP, APTT, PT), inflammatory indicators (WBC, CRP, PCT), RBC, Albumin, and clinical severity scores (SOFA, Caprini). Recursive feature elimination further indicated that a combination of the top six variables achieved the highest area under the curve (AUC) with minimal loss of predictive power. **Table 2** presents the distribution of selected variables across the training, test, and external validation cohorts. Significant inter−cohort differences were observed for FDP, PT, WBC, APTT, and CRP (*P* < 0.05), highlighting population heterogeneity and underscoring the need for robust external validation.

**Table 2:** Analysis of selected variables across the training, test, and external validation sets.

Variable	Training set, N = 513	Test set, N = 220	Internal validation set, N = 344	*P*-value
FDP	5.30 [1.89, 21.16]	4.77 [1.80, 12.65]	2.76 [1.90, 3.90]	<0.001
PT	14.80 [13.20, 16.50]	14.95 [13.50, 17.00]	14.50 [12.43, 16.00]	0.012
WBC	9.44 [7.81, 12.76]	8.48 [7.77, 12.11]	8.72 [5.82, 13.13]	<0.001
D-D	1.30 [0.50, 5.80]	1.70 [0.50, 6.62]	0.70 [0.50, 11.03]	0.306
APTT	39.60 [34.20, 45.20]	38.80 [33.28, 44.13]	37.90 [32.40, 42.70]	0.003
CRP	58.90 [10.00, 145.30]	38.70 [10.00, 129.00]	67.45 [19.28, 131.00]	0.030

Continuous variables are presented as median [IQR]. *P*-values were derived from the Kruskal-Wallis H test for overall comparisons among the three cohorts. For variables with statistically significant overall differences, pairwise comparisons were performed using Dunn’s *post-hoc* test with Bonferroni correction. FDP, fibrin degradation products; PT, prothrombin time; WBC, white blood cells; APTT, activated partial thromboplastin time; D-D, D-dimer; CRP, C-reactive protein.

### Comparative performance of machine learning models

**Figure 3:**
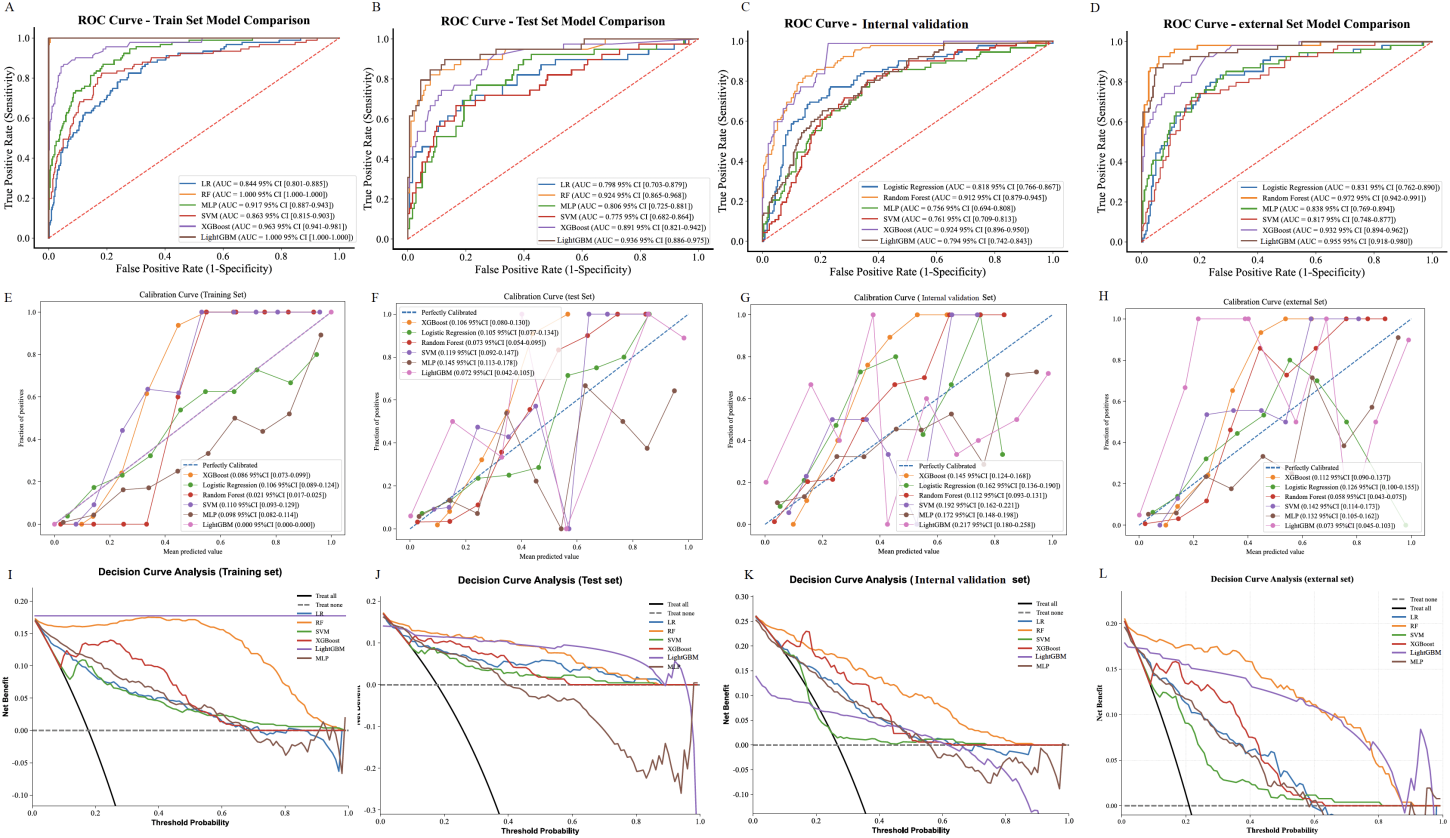
Evaluation of six prediction models on three data partitions (training, test, internal validation, external validation). Rows represent the three datasets, while columns show different performance metrics: ROC curves **(A–D)**, calibration plots **(E–H)**, and decision curve analysis **(I–L)**.

The predictive performance of six machine learning algorithms was evaluated across training, test, and external validation sets (**Figure 3**; **Table 3**).

**Table 3:** Comparison of prediction performance among different machine learning models on training, test, and external data.

Model	Dataset	Accuracy	Sensitivity	Precision	Specificity	F1 Score	AUC
SVM	Training	0.8538	0.1758	1	1	0.2991	0.8626
	Test	0.8409	0.1282	0.8333	0.9945	0.2222	0.775
	Internal validation	0.7384	0.0435	0.6667	0.9921	0.0816	0.7607
	External validation	0.8016	0.0741	0.8000	0.9951	0.1356	0.8170
XGBoost	Training	0.8655	0.2418	1	1	0.3894	0.9626
	Test	0.8409	0.1026	1	1	0.186	0.8908
	Internal validation	0.7471	0.0543	1	1	0.1031	0.9238
	External validation	0.8132	0.1111	1	1	0.2000	0.9320
LightGBM	Training	1	1	1	1	1	1
	Test	0.9136	0.641	0.8333	0.9724	0.7246	0.9364
	Internal validation	0.7616	0.2935	0.6136	0.9325	0.3971	0.7942
	External validation	0.9105	0.7222	0.8298	0.9606	0.7723	0.9546
Logistic Regression	Training	0.8558	0.3736	0.6667	0.9597	0.4789	0.8444
	Test	0.8773	0.4103	0.8	0.9779	0.5424	0.7983
	Internal validation	0.75	0.1739	0.6154	0.9603	0.2712	0.8181
	External validation	0.8249	0.3333	0.6667	0.9557	0.4444	0.8305
Random Forest	Training	0.9942	0.967	1	1	0.9832	0.9999
	Test	0.9136	0.5641	0.9167	0.989	0.6984	0.9238
	Internal validation	0.8314	0.4348	0.8696	0.9762	0.5797	0.9125
	External validation	0.9222	0.7037	0.9048	0.9803	0.7917	0.9718
MLP	Training	0.8616	0.7473	0.5862	0.8863	0.657	0.9165
	Test	0.7909	0.5128	0.4255	0.8508	0.4651	0.8061
	Internal validation	0.7442	0.4457	0.5256	0.8532	0.4824	0.7563
	External validation	0.8132	0.6481	0.5469	0.8571	0.5932	0.8379

95% confidence intervals for all metrics on the external validation cohort are provided in **Supplementary Table 3**.

Although XGBoost achieved a high external AUC of 0.9238, its external sensitivity was only 0.0543 and F1 score was 0.1031, indicating that the model predominantly predicted the negative class and failed to identify most true VTE cases. Such poor positive case detection renders the model clinically unusable for thromboprophylaxis decisions. LightGBM demonstrated perfect classification on the training set (AUC = 1.0) but showed substantial performance decay in external validation (AUC dropped to 0.7942, sensitivity 0.2935), indicating severe overfitting and limited generalizability.

In contrast, Random Forest exhibited the most balanced and robust performance. Key metrics supporting RF as the optimal model include: (1) Highest external AUC (0.9718), reflecting superior ability to discriminate between VTE and non−VTE cases in an independent cohort; (2) High internal validation accuracy (0.8314) and external accuracy (0.9222), indicating excellent overall prediction correctness; (3) Best F1 score in both internal (0.5797) and external (0.7917) validation, demonstrating an optimal trade−off between precision and recall in this imbalanced outcome setting; (4) Excellent generalization: training AUC (0.9999), internal validation AUC (0.9125), and external validation AUC (0.9718) showed consistent high performance with minimal decline, confirming effective control of overfitting and encouraging external validity in an independent institutional cohort. Therefore, Random Forest was selected as the final predictive model for subsequent interpretation and clinical application. Calibration metrics for all models in the external validation cohort are shown in **Supplementary Table 5**. The Random Forest model achieved the best calibration (intercept: −0.046, slope: 0.972, Brier: 0.058).

### Comparison with established clinical scoring systems

To further validate the incremental value of our model, we compared the Random Forest model with three established clinical scoring systems in the external validation cohort: the Wells score for DVT, the Caprini risk score, and the SOFA score. The RF model achieved an AUC of 0.9718 (95% CI: 0.957–0.987), significantly outperforming the Wells score (AUC 0.8431, 95% CI: 0.791–0.895), Caprini score (AUC 0.8492, 95% CI: 0.796–0.902), and SOFA score (AUC 0.8186, 95% CI: 0.763–0.874). DeLong tests confirmed that the RF model was superior to all three comparators (all *P* < 0.001). These findings confirm the superior discriminative performance of our ML−based approach over established VTE clinical scoring systems in the septic shock population. It is worth noting that some models with high AUC values, such as XGBoost, may still perform poorly at the default classification threshold due to inadequate calibration. This apparent discrepancy highlights that high AUC does not guarantee clinically useful performance at a fixed threshold, supporting our selection of Random Forest for its well−calibrated probability estimates.

### Statistical comparison between machine learning models

To formally compare the discriminative performance of the six machine learning algorithms, we performed DeLong tests on the external validation cohort. The Random Forest model achieved the highest AUC (0.9718) and significantly outperformed SVM (AUC 0.8170, *P* < 0.001), XGBoost (AUC 0.9320, *P* < 0.001), Logistic Regression (AUC 0.8305, *P* < 0.001), and MLP (AUC 0.8379, *P* < 0.001). There was no statistically significant difference between RF and LightGBM (AUC 0.9546, *P* = 0.31). However, LightGBM exhibited signs of overfitting (training AUC = 1.0 with performance decay in validation), whereas RF maintained consistent performance across all datasets with superior F1 score and calibration. Full pairwise comparisons are provided in **Supplementary Table 4**.

### Complementary role of conventional clinical scores to mitigate missed VTE

Given the low sensitivity (0.4348) of the Random Forest model in external validation, we further analysed the 23 false−negative cases (VTE occurred but model predicted low risk) to determine whether conventional clinical scores could serve as a backup screening tool. As shown in **Supplementary Table 1**, the majority of these false−negative patients had elevated SIRS (≥2, 82.6%) or SOFA (≥2, 91.3%) scores on ICU admission. Only one patient (4.3%) had both scores below these thresholds.

Based on this observation, we propose a two−step clinical workflow (**Supplementary Figure 1**): (1) Apply the RF model to all patients; (2) For those classified as low−risk by the model, automatically calculate SIRS and SOFA scores. If either score exceeds the threshold (SIRS ≥2 or SOFA ≥2), a clinical alert is triggered for further VTE evaluation. This complementary strategy would recapture 20 of 23 false−negative cases (86.9%), raising the effective “clinical sensitivity” to 0.8913, while maintaining high specificity.

### Model interpretability via SHAP analysis

**Figure 4:**
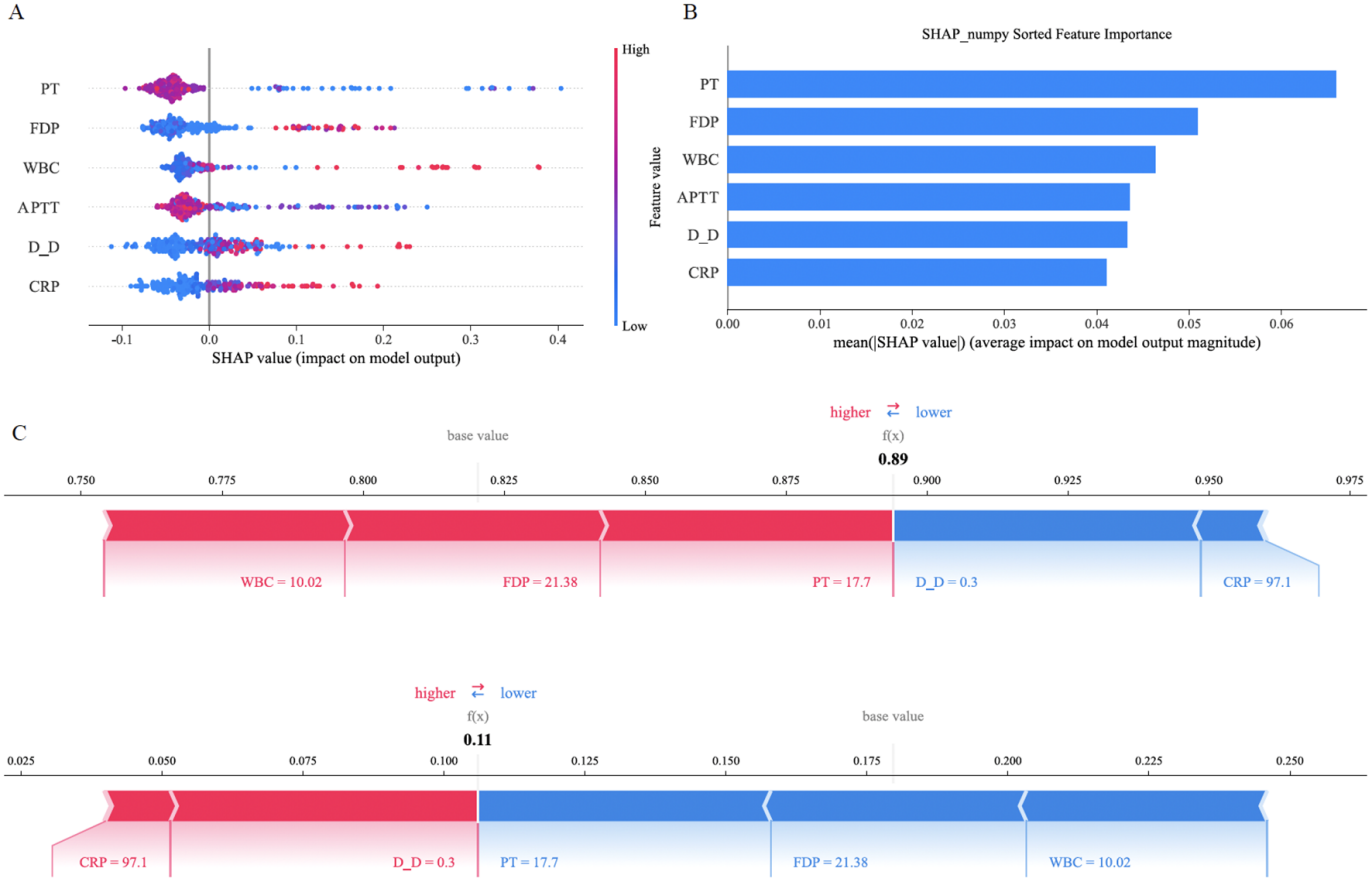
Interpretability analysis of the prediction model using SHAP. **(A)** SHAP summary plot: x−axis = SHAP value; y−axis = features; color gradient (red/blue) reflects original feature magnitude. **(B)** Logistic regression−based feature importance ranking derived from the absolute values of standardized coefficients. **(C)** Force plot visualization of prediction drivers for two independent cases.

To enhance clinical transparency, we applied SHAP to the Random Forest model. **Figure 4A** presents the SHAP summary plot, which visualizes the direction and magnitude of each feature’s contribution to VTE risk. The most influential predictors were PT, FDP, WBC, APTT, D-D, and CRP. Increased levels of FDP, WBC, D−D, and CRP, as well as shortened PT and APTT, showed positive correlations with a higher probability of VTE. **Figure 4B** shows the feature importance ranking derived from logistic regression for comparison, which exhibited a similar pattern, confirming the robustness of the selected variables. **Figure 4C** provides force plots for two representative patients, illustrating how individual feature values drive personalized risk predictions. This interpretable framework bridges the gap between model output and clinical intuition, facilitating individualized risk stratification in critically ill patients with septic shock.

## Discussion

In this retrospective cohort study, we successfully developed and externally validated an interpretable machine learning framework specifically tailored for predicting VTE in patients with septic shock. Among the six evaluated algorithms, the Random Forest model exhibited the most balanced and robust predictive performance, achieving the highest area under the curve (0.9125) and an optimal F1 score (0.5797) in the independent external validation cohort. By deploying the Boruta algorithm alongside recursive feature elimination, we distilled a parsimonious array of six critical predictors: FDP, PT, WBC, APTT, D-D, and CRP. Crucially, the integration of SHAP values transitioned our high-performing model from an opaque “black box” into a transparent tool that, after prospective validation, may provide individualized risk stratification to support clinical decision-making.

The application of machine learning in forecasting thrombotic events has accelerated significantly, yet predictive algorithms specifically conceptualized for the septic shock demographic remain conspicuously limited. For instance, while a comprehensive study by Zhang et al. (18) formulated a random forest algorithm to predict VTE within a generalized intensive care cohort across 207 centers, it did not isolate the unique pathophysiological milieu of septic shock. Furthermore, a recent systematic review of ML-based VTE prediction models revealed a persistent methodological bottleneck: although these algorithms frequently outperform traditional clinical scoring matrices, a pronounced majority lack external validation, thereby heavily curtailing their translational potential and reproducibility in diverse clinical environments (19). Our study effectively circumvents this limitation. By utilizing a temporally segregated external validation cohort, we demonstrated that the RF architecture maintained formidable discriminative power in both internal and external validation settings and avoided the severe overfitting phenomenon that undermined our LightGBM iteration.

Our methodological approach aligns with recent trends in explainable machine learning for clinical prediction. For instance, Cantaş Türkiş et al. developed robust and interpretable ML models for Alzheimer’s disease-related cognitive impairment using cerebrospinal fluid biomarkers, systematically comparing sparsity−based (LASSO), importance−based (Boruta), and consensus feature selection strategies (20). Their study demonstrated that Boruta−based models achieved higher sensitivity, while consensus−based models provided more balanced performance, with SHAP analyses highlighting biologically plausible biomarker contributions (20). Similarly, the same group developed an explainable AI−based clinical decision support system for predicting adverse outcomes in rhabdomyolysis, employing a comparable framework of ML model development, SHAP−based interpretability, and clinical utility evaluation (21). These studies, alongside ours, reflect a broader shift toward integrating rigorous feature selection, transparent model explanations, and calibration assessment in healthcare prediction modeling. Our study extends this paradigm to the septic shock population, specifically addressing the challenge of VTE prediction through external validation and a practical two−step workflow to mitigate sensitivity limitations.

From a pathophysiological perspective, the robustness of our model is underpinned by the biological plausibility of its selected parameters. The intimate crosstalk between systemic inflammation and the coagulation cascade, termed immunothrombosis, is the hallmark of sepsis-induced vascular dysfunction (22, 23). Our SHAP global summary confirmed that elevated levels of FDP, D-D, WBC, and CRP, in conjunction with abbreviated PT and APTT intervals, were the primary vectors driving VTE probability. D-D and FDP are recognized degradation byproducts that immediately signify an active, dysregulated thrombo-inflammatory state (24, 25). Interestingly, while previous meta-analyses generally correlate prolonged coagulation times with poor survival outcomes in progressive sepsis, our model captures a distinct, early hypercoagulable phenotype characterized by shortened PT and APTT (9). This suggests that the highest risk window for VTE in septic shock often occurs during the acute prothrombotic phase, prior to the onset of profound consumptive coagulopathy (26, 27). Concurrently, the selection of robust inflammatory indices (WBC and CRP) reinforces the paradigm that the magnitude of the immune response is inextricably coupled with thrombotic propensity (22, 23, 28).

The principal barrier to the bedside integration of complex machine learning algorithms is their inherent opacity, which historically limits clinician trust and hinders widespread adoption. By anchoring our RF model with SHAP analysis, we instituted a transparent, dual-tiered interpretive framework. On a macroscopic scale, the feature importance hierarchy corroborated conventional logistic regression outputs, ensuring baseline algorithmic reliability (29). On a microscopic, patient-specific level, the generation of individual force plots demystifies the predictive output, illustrating the directional and quantitative contribution of each laboratory parameter to a given prediction (12, 30). However, SHAP values reflect statistical associations derived from our cohort, not causal relationships. They should be interpreted as hypothesis−generating rather than prescriptive, and they do not replace clinical judgment. In the high-stakes environment of the ICU, where empirical anticoagulation carries substantial bleeding risks, this localized transparency may assist critical care physicians in evaluating personalized risk factors, but treatment decisions must remain grounded in comprehensive clinical assessment.

A legitimate concern regarding our RF model is its modest sensitivity (0.4348) in the internal validation cohort, which would result in more than half of true VTE cases being missed if used as a standalone screening tool at the default 0.5 threshold. This underscores that AUROC alone is insufficient for clinical evaluation. To address this, we assessed calibration (Brier score: 0.058; calibration slope: 0.972) and propose a two−step workflow combining the RF model with SIRS/SOFA scores as a safety net, raising effective sensitivity to 0.8913. Additionally, the classification threshold can be adjusted according to clinical context—lowering the threshold would increase sensitivity at the cost of more false positives, which may be acceptable in high−risk ICU settings. These considerations support a more balanced interpretation: while the model offers strong discriminative ability, it should be used as an adjunct to clinical judgment, not as a replacement.

The Random Forest model achieved a high training AUC of 0.9999, which raises a legitimate concern about potential overfitting. However, we believe this risk is well controlled for several reasons. First, Random Forest inherently mitigates overfitting through bootstrap aggregating and random feature selection, which reduce variance without increasing bias. Second, our hyperparameter configuration—particularly the limited tree depth (max_depth = 10) and relatively high min_samples_split (10)—was intentionally chosen to prevent trees from memorising noise in the training data. Third, the model’s performance in the independent external validation cohort (AUC 0.9718) showed only a modest decline from the training AUC (0.9999), which is substantially smaller than the decay observed for LightGBM (1.0 to 0.9546). This consistency across an entirely different institution strongly supports that the model has captured genuine clinical associations rather than spurious patterns. Nonetheless, we acknowledge that further validation in additional independent cohorts remains warranted to confirm the generalisability of our findings.

Notably, although several selected predictors (FDP, PT, WBC, APTT, and CRP) showed significant distributional differences across the derivation and external cohorts (**Table 1**), our RF model maintained robust discriminative performance and satisfactory calibration (Brier score: 0.058; calibration slope: 0.972) in the external set. We attribute this resilience to the tree−based algorithm’s inherent insensitivity to absolute value shifts across sites. Nevertheless, we acknowledge that site−specific recalibration of the model intercept may further enhance transportability when applied in new clinical settings. Importantly, while our model demonstrates strong predictive performance, the retrospective design precludes definitive conclusions about its clinical impact. Prospective implementation studies are needed to assess whether integration of this tool into clinical workflows improves patient outcomes, such as reducing time to appropriate thromboprophylaxis or lowering VTE incidence without increasing bleeding complications.

## Limitation

Several additional limitations warrant consideration. First, the two−step feature selection procedure (Boruta followed by RFE) was performed exclusively on the training set to avoid data leakage. While this approach reduces overfitting, it may introduce feature−selection bias, as the selected predictors may be optimized for this specific cohort and may not generalise to populations with different feature distributions. Second, the relatively low incidence of VTE (17.74%) created a class−imbalanced setting. Although Random Forest is inherently robust to class imbalance and we used balanced class weights, the model’s modest sensitivity (0.4348 in internal validation) reflects the difficulty of detecting a minority class event. This limitation is partially mitigated by the improved sensitivity in the external validation cohort (0.7037) and by the proposed two−step safety−net workflow (**Supplementary Figure 1**), but it remains a consideration for clinical use. Third, the reliance on default 0.5 threshold may not be optimal for this clinical context; threshold selection should be guided by the relative costs of missed VTE versus unnecessary anticoagulation, which may vary across clinical settings. Fourth, although the model was validated in an independent external cohort, both centres are located within the same province, limiting assessment of transportability to healthcare systems with different patient demographics, practice patterns, or laboratory standards. Multi−centre validation across geographically diverse populations is needed. Finally, the features were limited to baseline measurements within 24 hours of ICU admission; dynamic changes in inflammatory or coagulation markers over time were not captured, which may have additional predictive value.

## Conclusion

In conclusion, we established an interpretable, robust Random Forest-based VTE prediction model specifically tailored for critically ill patients with septic shock. Leveraging six readily accessible routine clinical parameters, this model demonstrates strong discriminative ability in both internal and external validation cohorts, with encouraging preliminary evidence of generalizability. Augmented by SHAP analysis, it facilitates understanding of model predictions by quantifying feature contributions. However, SHAP provides explanations of associations, not causal inference, and its clinical actionability requires prospective evaluation. This tool offers a transparent risk assessment framework that may assist in risk stratification for thromboprophylaxis after prospective validation, but should be used as an adjunct to, not a replacement for, clinical judgment.

## Data Availability

The raw data supporting the conclusions of this article will be made available by the authors, without undue reservation.
