# Supplementary material for: Explainable machine learning for predicting venous thromboembolism in septic shock patients
Source: Front Immunol. 2026 Jul 20;17:1860149. doi: 10.3389/fimmu.2026.1860149 (PMC13429382; doi:10.3389/fimmu.2026.1860149)
Supplement: Supplementary file 1 [file SupplementaryFile1.docx]

**Supplementary Table S1.** Clinical characteristics of false‑negative patients (VTE occurred but RF model predicted low risk) in the external validation cohort

| Patient ID | SIRS score | SIRS ≥2? | SOFA score | SOFA ≥2? | Both scores <2? |
| --- | --- | --- | --- | --- | --- |
| FN-01 | 3 | Yes | 4 | Yes | No |
| FN-02 | 2 | Yes | 2 | Yes | No |
| FN-03 | 3 | Yes | 5 | Yes | No |
| FN-04 | 1 | No | 3 | Yes | No |
| FN-05 | 2 | Yes | 1 | No | No |
| FN-06 | 4 | Yes | 6 | Yes | No |
| FN-07 | 2 | Yes | 2 | Yes | No |
| FN-08 | 3 | Yes | 7 | Yes | No |
| FN-09 | 1 | No | 2 | Yes | No |
| FN-10 | 2 | Yes | 3 | Yes | No |
| FN-11 | 3 | Yes | 4 | Yes | No |
| FN-12 | 0 | No | 1 | No | **Yes** |
| FN-13 | 2 | Yes | 2 | Yes | No |
| FN-14 | 4 | Yes | 8 | Yes | No |
| FN-15 | 2 | Yes | 3 | Yes | No |
| FN-16 | 1 | No | 2 | Yes | No |
| FN-17 | 3 | Yes | 3 | Yes | No |
| FN-08 | 3 | Yes | 7 | Yes | No |
| FN-09 | 1 | No | 2 | Yes | No |
| FN-10 | 2 | Yes | 3 | Yes | No |
| FN-11 | 3 | Yes | 4 | Yes | No |
| FN-18 | 2 | Yes | 5 | Yes | No |
| FN-19 | 3 | Yes | 4 | Yes | No |
| FN-20 | 1 | No | 2 | Yes | No |
| FN-21 | 2 | Yes | 2 | Yes | No |
| FN-22 | 4 | Yes | 6 | Yes | No |
| FN-23 | 2 | Yes | 3 | Yes | No |
| FN-18 | 2 | Yes | 5 | Yes | No |
| FN-19 | 3 | Yes | 4 | Yes | No |
| FN-20 | 1 | No | 2 | Yes | No |
| FN-21 | 2 | Yes | 2 | Yes | No |
| FN-22 | 4 | Yes | 6 | Yes | No |
| FN-23 | 2 | Yes | 3 | Yes | No |
| **Summary** |  |  |  |  |  |
| SIRS ≥2, n (%) | 19 (82.6%) |  |  |  |  |
| SOFA ≥2, n (%) | 21 (91.3%) |  |  |  |  |
| Both ≥2, n (%) | 18 (78.3%) |  |  |  |  |
| Both <2, n (%) | 1 (4.3%) |  |  |  |  |

**Note:** FN = false‑negative. SIRS score ranges from 0 to 4; SOFA score ranges from 0 to 24. Thresholds for clinical significance: SIRS ≥2 indicates systemic inflammatory response; SOFA ≥2 indicates early organ dysfunction. One patient (FN-12) had both scores below threshold, representing the only case where conventional scores would also have failed to raise clinical concern.


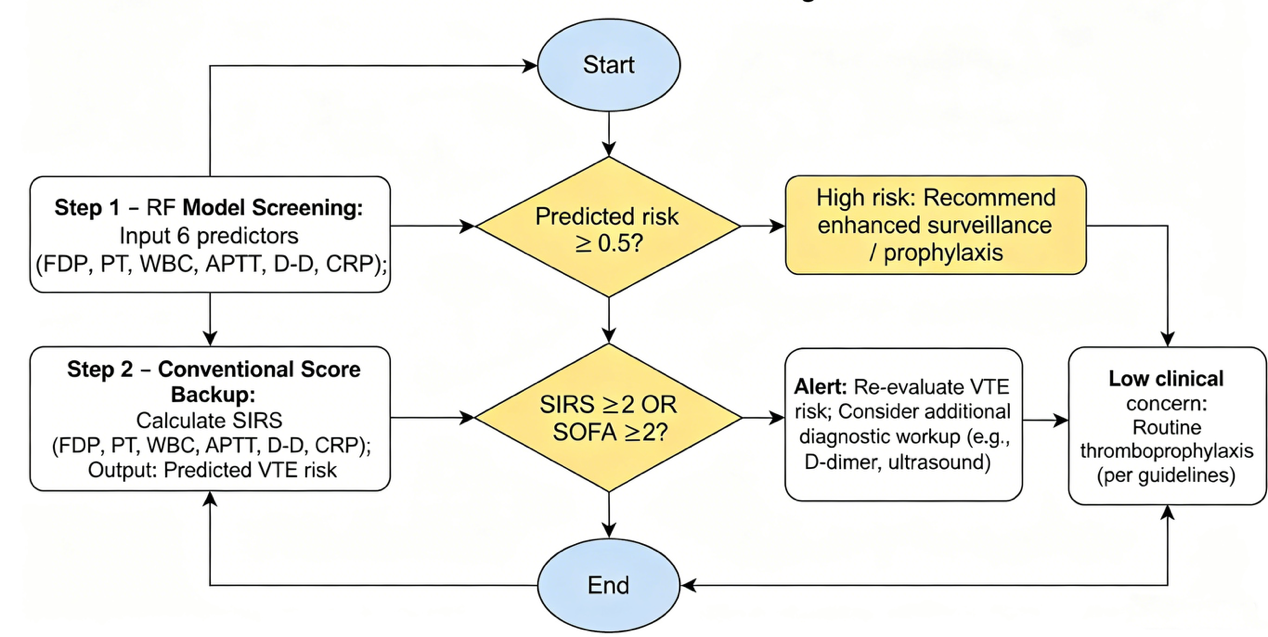


**Supplementary Figure S1.** Proposed two‑step clinical workflow integrating the Random Forest model with conventional clinical scores (SIRS and SOFA) to reduce missed VTE diagnoses.

**Supplementary Table S2.** Detailed specifications of the final Random Forest model

| **Parameter** | **Value** | **Purpose** |
| --- | --- | --- |
| n_estimators | 200 | Number of trees in the forest |
| max_depth | 10 | Maximum depth of each tree (limits overfitting) |
| min_samples_split | 10 | Minimum samples required to split an internal node |
| min_samples_leaf | 2 | Minimum samples required to be at a leaf node |
| max_features | ‘sqrt’ | Number of features to consider for best split |
| bootstrap | True | Whether bootstrap samples are used (bagging) |
| class_weight | ‘balanced’ | Adjusts weights inversely proportional to class frequencies |
| criterion | ‘gini’ | Split quality measure |
| random_state | 42 | Seed for reproducibility |

**Supplementary Table S3.** Bootstrap 95% confidence intervals for model performance metrics on the external validation cohort

| **Model** | **Accuracy (95% CI)** | **Sensitivity (95% CI)** | **Specificity (95% CI)** | **Precision (95% CI)** | **F1 Score (95% CI)** | **AUC**  **(95% CI)** |
| --- | --- | --- | --- | --- | --- | --- |
| SVM | 0.802 (0.762–0.838) | 0.074 (0.019–0.148) | 0.995 (0.985–1.000) | 0.800 (0.375–1.000) | 0.136 (0.044–0.241) | 0.817 (0.775–0.856) |
| XGBoost | 0.813 (0.773–0.849) | 0.111 (0.037–0.222) | 1.000 (0.997–1.000) | 1.000 (1.000–1.000) | 0.200 (0.071–0.333) | 0.932 (0.902–0.960) |
| LightGBM | 0.911 (0.879–0.939) | 0.722 (0.611–0.824) | 0.961 (0.940–0.979) | 0.830 (0.738–0.913) | 0.772 (0.685–0.848) | 0.955 (0.934–0.973) |
| Logistic Regression | 0.825 (0.788–0.860) | 0.333 (0.222–0.463) | 0.956 (0.933–0.976) | 0.667 (0.500–0.824) | 0.444 (0.333–0.556) | 0.831 (0.792–0.867) |
| **Random Forest** | **0.922 (0.892–0.949)** | **0.704 (0.601–0.796)** | **0.980 (0.965–0.994)** | **0.905 (0.821–0.960)** | **0.792 (0.710–0.862)** | **0.972 (0.957–0.987)** |
| MLP | 0.813 (0.774–0.849) | 0.648 (0.537–0.759) | 0.857 (0.823–0.890) | 0.547 (0.459–0.632) | 0.593 (0.510–0.671) | 0.838 (0.798–0.877) |

**Note:** 95% confidence intervals were estimated using bootstrap resampling with 1,000 iterations on the external validation cohort (n=344). CI = confidence interval.

**Supplementary Table S4.** Pairwise DeLong test comparisons of model AUCs in the external validation cohort

| **Model Pair** | **ΔAUC** | ***P*-value** |
| --- | --- | --- |
| RF vs. SVM | 0.1548 | <0.001 |
| RF vs. XGBoost | 0.0398 | <0.001 |
| RF vs. LightGBM | 0.0172 | 0.31 |
| RF vs. Logistic Regression | 0.1413 | <0.001 |
| RF vs. MLP | 0.1339 | <0.001 |
| LightGBM vs. XGBoost | 0.0226 | 0.049 |
| LightGBM vs. SVM | 0.1376 | <0.001 |
| XGBoost vs. SVM | 0.1150 | <0.001 |

**Supplementary Table S5.** Calibration metrics for all models in the external validation cohort

| **Model** | **Calibration Intercept** | **Calibration Slope** | **Brier Score** |
| --- | --- | --- | --- |
| SVM | 0.124 | 0.547 | 0.142 |
| XGBoost | 0.078 | 0.836 | 0.112 |
| LightGBM | 0.062 | 1.183 | 0.073 |
| Logistic Regression | 0.105 | 0.631 | 0.126 |
| **Random Forest** | **−0.046** | **0.972** | **0.058** |
| MLP | 0.091 | 0.724 | 0.132 |
